# Identification of prostate cancer specific methylation biomarkers from a multi-cancer analysis

**DOI:** 10.1186/s12859-021-04416-w

**Published:** 2021-10-12

**Authors:** Yiyi Pu, Chao Li, Haining Yuan, Xiaoju Wang

**Affiliations:** School of Bioengineering, Hangzhou Medical College, No. 182 Tianmushan Road, Hangzhou, 310013 Zhejiang Province People’s Republic of China

**Keywords:** DNA methylation, Diagnosis, Biomarker, Prostate cancer, Bioinformatics

## Abstract

**Background:**

Detecting prostate cancer at a non-aggressive stage is the main goal of prostate cancer screening. DNA methylation has been widely used as biomarkers for cancer diagnosis and prognosis, however, with low clinical translation rate. By taking advantage of multi-cancer data from The Cancer Genome Atlas (TCGA) and Gene Expression Omnibus (GEO), we aimed to identify prostate cancer specific biomarkers which can separate between non-aggressive and aggressive prostate cancer based on DNA methylation patterns.

**Results:**

We performed a comparison analysis of DNA methylation status between normal prostate tissues and prostate adenocarcinoma (PRAD) samples at different Gleason stages. The candidate biomarkers were selected by excluding the biomarkers existing in multiple cancers (pan-cancer) and requiring significant difference between PRAD and other urinary samples. By least absolute shrinkage and selection operator (LASSO) selection, 8 biomarkers (cg04633600, cg05219445, cg05796128, cg10834205, cg16736826, cg23523811, cg23881697, cg24755931) were identified and in-silico validated by model constructions. First, all 8 biomarkers could separate PRAD at different stages (Gleason 6 vs. Gleason 3 + 4: AUC = 0.63; Gleason 6 vs. Gleason 4 + 3 and 8–10: AUC = 0.87). Second, 5 biomarkers (cg04633600, cg05796128, cg23523811, cg23881697, cg24755931) effectively detected PRAD from normal prostate tissues (AUC ranged from 0.88 to 0.92). Last, 6 biomarkers (cg04633600, cg05219445, cg05796128, cg23523811, cg23881697, cg24755931) completely distinguished PRAD with other urinary samples (AUC = 1).

**Conclusions:**

Our study identified and in-silico validated a panel of prostate cancer specific DNA methylation biomarkers with diagnosis value.

**Supplementary Information:**

The online version contains supplementary material available at 10.1186/s12859-021-04416-w.

## Background

For men, prostate cancer is the second most common cancer and the fifth most deadly cancer worldwide [1], which has been a long-term threat to men's health. Because early-stage prostate cancer has no symptom, it is often found at an advanced stage. Therefore, the main goal of prostate screening is to identify disease at a curable stage [2]. Prostate specific antigen (PSA) is a protein produced by prostate gland and based on elevated blood PSA level in prostate cancer patients, PSA screening was developed. Several studies in 1990s showed PSA screening could improve localized prostate cancer detection and had the potential to decrease disease mortality [3, 4]. However, the improvement by PSA screening is not without cost. Because several abnormal conditions of prostate can elevate PSA level, high over-diagnosis rate of PSA test varying from 1.7% to 67% were reported [5]. In recent guidelines issued by the United States Preventive Medicine Task Force (USPSTF), PSA screening was not recommended for men over 70 years old, and regular PSA screening required caution and discussion with clinician for men between 55 and 69 years old [6].

DNA methylation is one of the main epigenetic modifications with great potential for biomarker development [7]. Many genes were found as potential biomarkers based on their methylation status for risk prediction, diagnosis, prognosis and treatment response in various cancer types [8]. Although a large number of differentially methylated genes were identified, only 14 of them have been translated into clinical tests [9], highlighting the urgent need for further development of methylation biomarkers.

In this study, we aimed to identify prostate cancer specific biomarkers with the ability to separate between non-aggressive and aggressive prostate tumors based on their DNA methylation levels. By taking advantage of the methylation data from public databases, such as TCGA and GEO, we identified a panel of DNA methylation biomarkers specific for prostate tumor, which have potential diagnosis value.

## Methods

All critical R scripts were in Additional file 7.

### Data collection

All cancer types with more than 5 normal samples and metastatic information were selected from TCGA (https://portal.gdc.cancer.gov/). DNA Methylation data (Illumina Human Methylation 450) for 16 main cancer subtypes were downloaded for this study: bladder urothelial carcinoma (BLCA), breast invasive ductal carcinoma (D_BRCA), breast invasive lobular carcinoma (L_BRCA), colon adenocarcinoma (COAD), esophageal adenocarcinoma (ESCA), head and neck squamous cell carcinoma (HNSC), renal clear cell carcinoma (KIRC), renal papillary cell carcinoma (KIRP), hepatocellular carcinoma (LIHC), lung adenocarcinoma (LUAD), lung squamous cell carcinoma (LUSC), pancreatic adenocarcinoma (PAAD), prostate adenocarcinoma (PRAD), rectal adenocarcinoma (READ), follicular thyroid carcinoma (F_THCA) and papillary thyroid carcinoma (P_THCA).

The probe annotation file was downloaded from GEO (GPL13534, HumanMethylation450_15017482, Illumina Inc.). Clinical data were downloaded from cBioPortal for Cancer Genomics (http://www.cbioportal.org/) [10] by its web API.

### Data processing

In AJCC CANCER STAGING MANUAL, tumor node metastasis (TNM) system is used as a general criterion to classify cancers by size and extent of the primary tumor (T), involvement of regional lymph node (N), and presence or absence of distant metastases (M) [11]. Based on TNM system, tumor samples in our study were classified into two main groups based on their metastasis state: localized tumors (N0 and M0: No regional lymph node metastases and No distant metastases), metastatic tumors (Regional lymph node metastases or Distant metastases or both). All NM information we referred to were pathological ones (stage at sample collection), except for PRAD which only had clinical M (stage at diagnosis).

Methylation levels were measured as β values for all 485,577 sites. β value, calculated as the ratio of methylated probe signal and total probe signal, ranges from 0 (entirely unmethylated) to 1 (entirely methylated). M value, calculated as log2 ratio of methylated probe signal and unmethylated probe signal, can be transferred from β value by Eq. (1). M values were found to provide better performance in differential methylation analyses [12] and thus were used in our methylation analyses and model construction.

1 $$M = \log 2\left( {\frac{\beta }{1 - \beta }} \right)$$

For each cancer type, probes with missing data in normal or tumor samples were removed. Meanwhile, cross-reactive probes (probes that hybridize to alternate sequences) [13], probes with certain genomic factors (e.g. SNPs and INDELs) [14] and probes located on sex chromosomes were also removed.

### Data analysis and pan-cancer biomarker selection

R package ‘limma’ (3.30.13) [15] was used to compare among normal samples (N), localized tumor samples (LT) and metastatic tumor samples (MT) for all 16 cancer types respectively based on their M values. Potential biomarkers for each cancer type were selected based on the significance of difference between N and LT (FDR < 0.05) and between N and MT (FDR < 0.05). The potential pan-cancer biomarkers were defined as biomarkers existing among more than half of cancer types (> 8) and exhibiting the same variation trend from N to LT and from N to MT. Results of this part were in Additional file 1: Table S1.

### Prostate cancer specific biomarker selection

All 484 PRAD samples were classified into five groups based on Gleason scores. Potential PRAD specific biomarkers were identified based on the following criteria:

- Significant methylation difference between normal and all PRAD Gleason groups (FDR < 0.05);
- Significant methylation difference among PRAD Gleason groups (FDR < 0.05);
- Significant methylation difference between prostate cancer samples and whole set of urinary system related samples (normal prostate, normal bladder, normal kidney, BLCA, KIRC, KIRP) (FDR < 0.05) and detectable mean β difference (> 0.1);

From potential PRAD specific biomarkers, we further excluded pan-cancer biomarkers. As for biomarkers with similar methylation profile, we randomly selected one to keep the Pearson correlation coefficient (R) of all biomarker pairs lower than 0.8. Main results of this part were in Additional file 2: Table S2, Additional file 3: Table S3 and Additional file 4: Table S4.

### LASSO selection and model construction

R package ‘lars’ (1.2) was used to further refine the list of potential biomarkers by LASSO penalization. In order to obtain reliable results, bootstrap method was performed: each time, a dataset with the same sample size as the original one was randomly selected with replacement to build a LASSO model. Within all bootstrapped datasets, sites with non-zero coefficients in more than 99% LASSO models were kept. Results of this part were in Additional file 5: Table S5.

To measure the predictive ability of biomarkers in separating multiple groups of samples, generalized linear models were constructed based on PRAD dataset from TCGA. We repeated the following process 1000 times and calculated the area under the curve (AUC) of receiver operating characteristic curve (ROC): randomly selected methylation data (M values) of 70% samples to train a model and the rest 30% samples for validation. Results of this part were in Additional file 6: Table S6.

Four datasets from GEO were downloaded and used for in-silico validation: GSE47915, GSE76938, GSE112047 and GSE52955. All four GEO datasets were based on the same platform (Illumina Human Methylation 450) and sample compositions were as follows: GSE47915 (4 Gleason-6 prostate tumors and 4 benign prostate tissues), GSE76938 (63 normal tissues and 73 PRAD), GSE112047 (16 normal tissues and 31 PRAD), GSE52955 (Kidney: 6 normal and 17 tumor samples; Bladder: 5 normal and 25 tumor samples; Prostate: 5 normal and 25 tumor samples). Generalized linear models were constructed based on the whole PRAD dataset from TCGA and validated by GEO datasets. In order to construct an effective model using the least critical biomarkers, we selected the model with the lowest Akaike Information Criterion (AIC).

## Results

### Identification of pan-cancer biomarkers

**Fig. 1:**
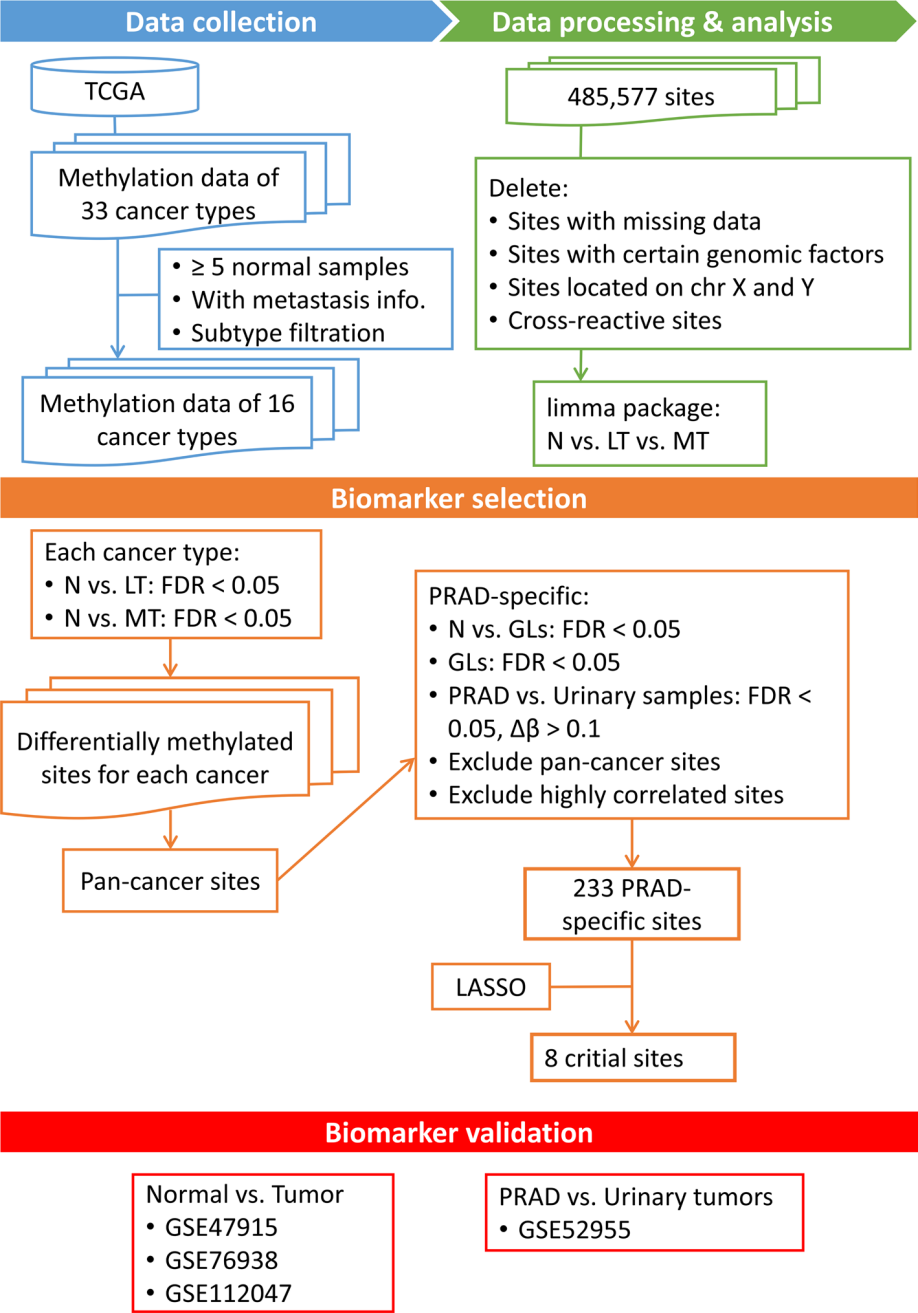
Flowchart of our biomarker identification and preliminary validation

In order to select prostate cancer specific biomarkers, we first identify pan-cancer biomarkers for further exclusion. We took advantage of tumor node metastasis (TNM) information and divided the samples into localized and metastatic tumors. The localized tumors here were defined as tumors with neither regional lymph node metastases nor distant metastases. The metastatic tumors here were defined as tumors from primary sites which have regional lymph node metastases or distant metastases. We then performed a multi-comparison for normal tissues, localized tumors and metastatic tumors based on a linear model. Genomic sites with significant methylation difference between normal tissues and localized tumors (FDR < 0.05), and also between normal tissues and metastatic tumors (FDR < 0.05) were considered as the candidate biomarkers. We identified a large list of methylation biomarkers for each cancer type. The number of biomarkers varied from 16,595 for LIHC to 186,944 for LUSC. After combining the biomarkers from all cancers, we finally identified 17,969 hyper-methylated (methylation level of tumors higher than normal tissues) and 16,527 hypo-methylated pan-cancer biomarkers. The pan-cancer biomarker selection procedure was summarized in Fig. 1.

### Identification of prostate cancer specific biomarkers

For prostate cancer, Gleason scores is one commonly used system to grade prostate cancer by the histological appearance of carcinoma cells [16]. Given the prognosis difference between Gleason score 3 + 4 and 4 + 3, a five-grade new system was proposed [17]. Therefore, we first divided all prostate tumors from TCGA into five groups (Gleason score 6, Gleason score 3 + 4, Gleason score 4 + 3, Gleason score 8, Gleason scores 9–10). After multi-comparison between normal and tumors from all five groups, only one significantly differential methylation site between Gleason 6 and 3 + 4 (cg18554116, FDR = 0.023) and one between Gleason 4 + 3 and 8 (cg13614962, FDR = 0.039) were identified. Therefore, we merged groups with similar methylation profile and reclassified prostate tumors into three Gleason groups (GL 1: Gleason score = 6 and 3 + 4, GL 2: Gleason score 4 + 3 and 8, GL 3: Gleason scores 9 and 10). A total of 3542 potential sites were identified with significant differences (FDR < 0.05) between normal tissues and three GL groups, and more importantly, among all three GL groups. We further applied the selection criteria described in Methods, and finally identified a total of 263 prostate tumor specific biomarkers.

**Fig. 2:**
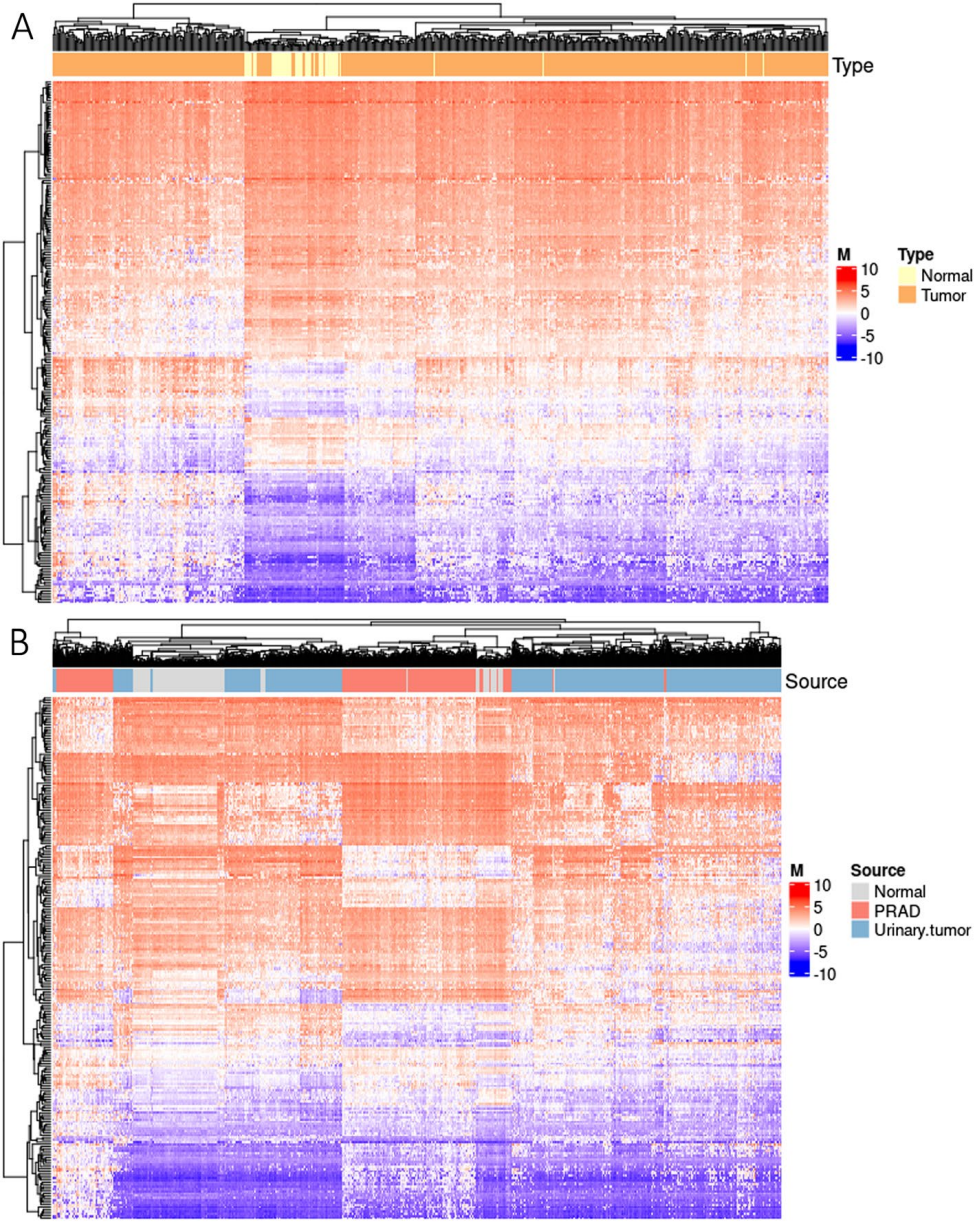
Methylation level (M values) of 233 prostate cancer specific biomarkers in TCGA datasets. **a** M values of 233 biomarkers in 533 prostate related samples (PRAD and normal samples). **b** M values of 233 biomarkers in 1749 samples. ‘Normal’ includes normal samples from bladder, kidney and prostate. ‘Urinary.tumor’ includes BLCA, KIRC and KIRP

To avoid the redundancy introduced by the highly similar methylation profiles, we performed a correlation analysis between all site pairs. We finally selected 233 sites (out of 263) to keep the Pearson correlation coefficient (R) among all site pairs lower than 0.8. Heatmaps of selected sites’ methylation level (M values) indicated their ability in separating normal versus PRAD (Fig. 2a) and PRAD versus urinary samples (Fig. 2b).

**Fig. 3:**
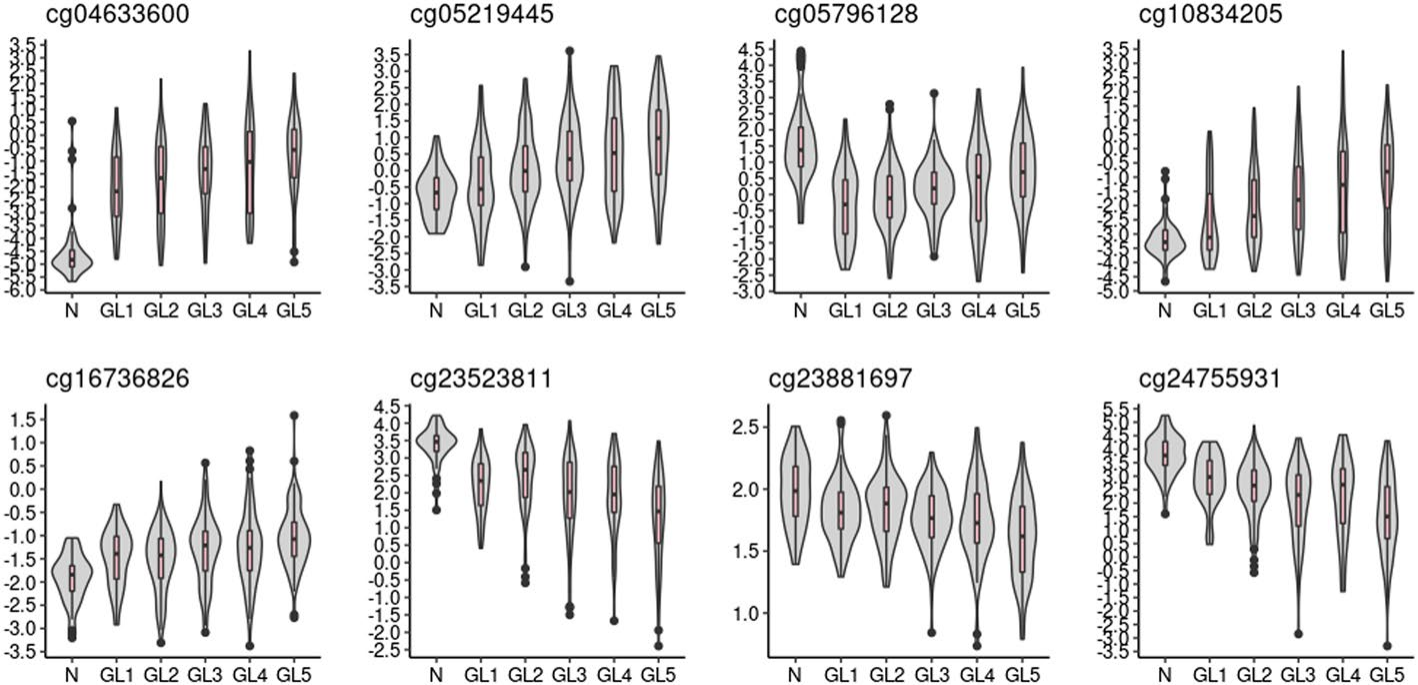
Methylation levels (M values) of all 8 selected PRAD-specific biomarkers. M values of normal tissues and five Gleason groups are shown (N: normal tissues, GL1: Gleason 6 prostate tumors, GL2: Gleason 3 + 4 prostate tumors, GL3: Gleason 4 + 3 prostate tumors, GL4: Gleason 8 prostate tumors, GL5: Gleason 9–10 prostate tumors)

LASSO penalization was further applied to select a panel of most valuable sites. 1000 bootstrapped datasets were produced by TCGA PRAD dataset (each time a dataset with the same sample size as the original one was randomly selected with replacement) to build a LASSO model. 8 sites (cg04633600, cg05219445, cg05796128, cg10834205, cg16736826, cg23523811, cg23881697, cg24755931) with non-zero coefficients in > 99% LASSO models were selected as our final PRAD specific biomarkers (Fig. 3). 6 out of these 8 sites were located within 6 gene regions (*SLCO4C1, TBC1D1, EDN2, GUCY2C, EHD1, CDC42BPB*) respectively. The whole biomarker selection procedure was summarized in Fig. 1.

### Predictive ability of the prostate cancer specific biomarkers

Based on LASSO penalization and bootstrap method, a total of 8 biomarkers (cg04633600, cg05219445, cg05796128, cg10834205, cg16736826, cg23523811, cg23881697, cg24755931) were identified with the potential to separate multiple Gleason prostate tumors, normal versus prostate tumors and prostate tumors versus urinary system samples.

1. Ability of separating multiple Gleason prostate tumors
  Due to the lack of Gleason information of public available dataset, random sampling validation method was used to evaluate the model. To detect early-stage prostate cancer before metastasis and with the notice of similar methylation profiles between Gleason 6 and Gleason 3 + 4 samples, we classified tumor samples into three groups (GL1: Gleason score = 6, GL2: Gleason score = 3 + 4, GL3: higher Gleason score prostate tumors). For 1000 times, 70% data was randomly used to construct a generalized linear model and the rest 30% data for validation. In order to test the predictive ability of our biomarkers, we tested models built by three types of information: (1) 8 sites + PSA + age; (2) 8 sites; (3) PSA + age. Using the above method, we tested the ability of our model in predicting GL1 versus GL2 (Fig. 4a) and GL1 versus GL3 (Fig. 4b). As we expected, models had higher predictive ability in GL1 versus GL3 than GL1 versus GL2. Although GL1 and GL2 had similar methylation profiles, the model built by methylation level of 8 sites with/without extra information still performed significantly better than model only based on PSA and age (*p* < 0.0001). Our analysis unexpectedly showed that model built by 8 sites without PSA and age information had significant higher AUC than model with such information (*p* < 0.0001).

**Fig. 4:**
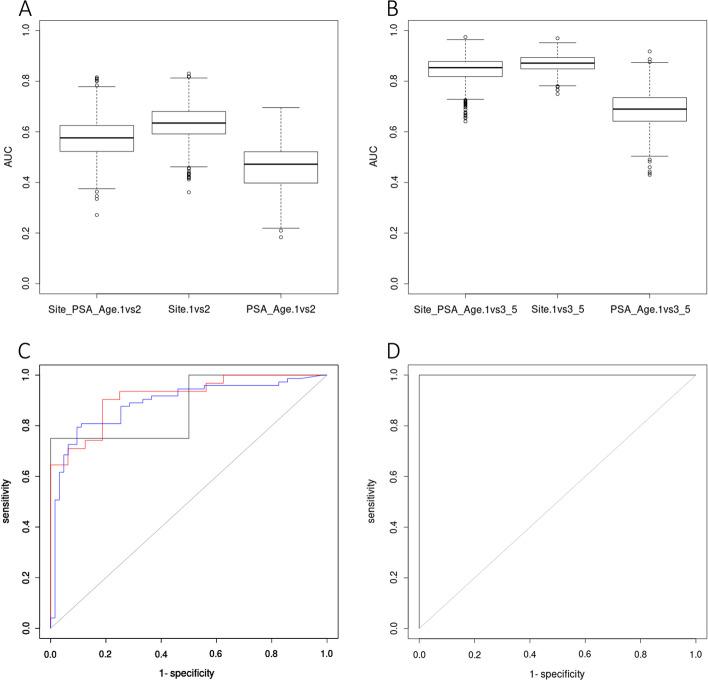
Model construction results of prostate cancer specific biomarkers. **a** AUC distribution of models in separating Gleason 6 and Gleason 3 + 4 prostate tumors. Models built by 8 biomarkers + PSA + age resulted in AUCs 0.57 ± 0.005. Models built by 8 biomarkers resulted in AUCs 0.63 ± 0.004. Models built only by PSA + age resulted in AUCs 0.46 ± 0.005. **b** AUC distribution of models in separating Gleason 6 with Gleason 4 + 3 and 8–10 prostate tumors. Models built by 8 biomarkers + PSA + age resulted in AUCs 0.84 ± 0.003. Models built by 8 biomarkers resulted in AUCs 0.87 ± 0.002. Models built only by PSA + age resulted in AUCs 0.67 ± 0.004. **c** ROC of three validating GEO datasets in separating normal tissue and prostate tumors: GSE47915 in black (AUC = 0.88), GSE76938 in blue (AUC = 0.89), GSE112047 in red (AUC = 0.92). D) ROC of one GEO dataset in separating prostate tumors and other urinary related samples: GSE52955 (AUC = 1)
2. Ability of separating normal tissues and prostate tumors
  Based on our biomarker panel, we used the whole TCGA PRAD datasets to build a generalized linear model by 5 biomarkers with the lowest AIC to separate normal tissues and PRAD samples, 0.878 * cg04633600 − 0.722 * cg05796128 − 0.853 * cg23523811 − 1.524 * cg23881697 − 0.964 * cg24755931 + 13.851. To validate the discriminatory power of the model in distinguishing prostate tumors from normal tissues in-silico, three GEO datasets were included (GSE47915: 4 Gleason-6 prostate tumors and 4 benign prostate tissues; GSE76938: 63 normal and 73 PRAD; GSE112047:16 normal and 31 PRAD) to draw the ROC (Fig. 4c). It turned out that our model can effectively separate normal tissues with prostate tumors (AUC ranging from 0.88 to 0.92).
3. Ability of separating prostate tumors and other urinary samples
  Clinical relevant biomarkers are expected to be prostate cancer specific, especially among other urinary related cancers. Therefore, we built a generalized linear model for two groups of samples, PRAD and urinary samples (BLCA, KIRC, KIRP, normal bladder, normal kidney and normal prostate). Based on significant differences between PRAD and other urinary samples, a model using 6 biomarkers, −0.745 * cg04633600 − 0.864 * cg05219445 + 1.818 * cg05796128 + 0.899 * cg23523811 − 1.355 * cg23881697 + 0.518 * cg24755931 − 7.394, could completely separate the prostate tumors from other urinary related samples in GSE52955 (Kidney: 6 normal and 17 tumor samples; Bladder: 5 normal and 25 tumor samples; Prostate: 5 normal and 25 tumor samples) with AUC = 1 (Fig. 4d).

## Discussion

While a large number of differentially methylated genes were identified, in prostate cancer the only commercially available methylation-based test is ‘ConfirmMDx’ (MDxHealth, Inc, Irvine, CA), which relies on the methylation status of three genes (GSTP1, APC and RASSF1) from biopsy tissues to avoid unnecessary repeat biopsies [18]. In addition to ConfirmMDx, numerous publications also identified differentially methylated genes from public databases [19–21]. With HumanMethylation450 array data from public databases, we are able to analyze methylation profile of both intragenic and intergenic regions. In all 485,577 sites tested by HumanMethylation450 array, there are about 25% sites in intergenic region. In order to identify the undiscovered biomarkers, our analysis took intergenic sites into account and identified genome-wide methylation sites as the potential biomarkers.

Public databases like TCGA provided a great opportunity for multi-cancer analysis. Most of the peer-reviewed literatures simply divided the patient samples into normal tissues and tumors and identified the genes with different methylation level between those two groups as potential biomarkers [22–25]. Due to the dynamic features of methylation profile in cancer stages [26], in our pan-cancer biomarker selection, we innovatively divided the TCGA tumor samples into localized tumors and metastatic tumors based on TNM information. After comparing the DNA methylation status between normal prostate tissues and prostate adenocarcinoma (PRAD) samples within different Gleason groups, we identified 8 potential biomarkers which could separate PRAD within different Gleason groups. 6 out of 8 biomarkers located in gene regions. As we expected, all 6 related genes have not been identified as biomarker before which was mainly due to our selection procedure. In our study, we no longer treated differential methylation level of whole gene or whole CpG island as marker. Instead, we zoomed in to analyze the methylation level of CpG sites. Therefore, by our method, we were able to identify sites within genes that are not differentially methylated.

Due to similar methylation profiles between Gleason 6 and Gleason 3 + 4 prostate tumors, the predictive ability of our biomarker panel was not high. However, our panel of biomarkers still performed significantly better than PSA screening. Together with the results that our biomarkers can effectively separate Gleason 6 with Gleason 4 + 3 and 8–10 prostate tumors, our biomarkers were still very promising in separating non-aggressive with aggressive prostate tumors.

Urine has been reported as a perfect medium to detect the molecular biomarker for prostate cancer. For example, O'reilly et al. combined a DNA methylation panel of previously reported genes (*GSTP1, SFRP2, IGFBP3, IGFBP7, APC,* and *PTGS2*) to detect high-risk prostate tumors by urine samples [27]. Similarly, Zhao et al. developed a urinary methylation assay based on methylation level of two genes (*HOXD3* and *GSTP1*) [28] and Bakavicius et al. combined PSA test and urinary methylation tests of three genes (RARB, RASSF1, GSTP1) [29]. However, the technique of qMSP, which is commonly used in previous studies to measure the general methylation of gene region, requires aberrant methylation in CpG islands [30]. In our current study, we identified the methylation sites within both CpG islands and non-CpG islands regions. Therefore, pyrosequencing [31], a technique to quantify single loci methylation level, could warrant our results for future clinical application. Indeed Yao et al. demonstrated pyrosequencing could effectively detect the methylation difference of loci cg05163709 in patient urine samples [32]. Together with the ability of our biomarkers in separating PRAD with other urinary samples, our panel of biomarkers showed great potential in urine test development.

## Conclusions

In summary, this study identified and in-silico validated 8 methylation-based biomarkers which were valuable for aggressive prostate cancer detection.

## Supplementary Information

**Additional file 1**. Results of pan-cancer sites.

**Additional file 2**. Results of comparison analysis among five Gleason score tumors.

**Additional file 3**. Results of comparison analysis among normal tissues and three Gleason score tumors.

**Additional file 4**. Results of comparison analysis between PRAD and other urinary samples.

**Additional file 5**. Results of LASSO selection.

**Additional file 6**. AUCs of model using all 8 biomarkers, PSA and age.

**Additional file 7**. Critical R scripts.

## Data Availability

All data generated or analyzed during this study are included in this published article and its supplementary information files.

## Abbreviations

AIC: Akaike information criterion
AUC: Area under the curve
BLCA: Bladder urothelial carcinoma
COAD: Colon adenocarcinoma
D_BRCA: Breast invasive ductal carcinoma
ESCA: Esophageal adenocarcinoma
F_THCA: Follicular thyroid carcinoma
GEO: Gene expression omnibus
HNSC: Head and neck squamous cell carcinoma
KIRC: Renal clear cell carcinoma
KIRP: Renal papillary cell carcinoma
LASSO: Least absolute shrinkage and selection operator
LIHC: Hepatocellular carcinoma
LT: Localized tumor samples
LUAD: Lung adenocarcinoma
LUSC: Lung squamous cell carcinoma
L_BRCA: Breast invasive lobular carcinoma
N: Normal samples
MT: Metastatic tumor samples
PAAD: Pancreatic adenocarcinoma
PRAD: Prostate adenocarcinoma
PSA: Prostate specific antigen
P_THCA: Papillary thyroid carcinoma
qMSP: Quantitative methylation-specific polymerase chain reaction
READ: Rectal adenocarcinoma
ROC: Receiver operating characteristic curve
TCGA: The cancer genome atlas
TNM: Tumor node metastasis
