# Supplementary material for: Identification of prostate cancer specific methylation biomarkers from a multi-cancer analysis
Source: BMC Bioinformatics. 2021 Oct 12;22:492. doi: 10.1186/s12859-021-04416-w (PMC8507340; doi:10.1186/s12859-021-04416-w)
Supplement: Supplementary file 7 — Additional file 7. Critical R scripts. [file 12859_2021_4416_MOESM7_ESM.pdf]

TCGA_BLCA_process.R

#Script to apply comparison analysis between normal tissues and localized/metastastic tumors by limma for each cancer type, taking BLCA as an example

```
library(data.table)
library(limma)

##original files----------------------------------------------------------------------------------
origin_matrix <- fread("BLCA/beta_matrix_final",header = T,sep = "\t")
probes <- read.table("probes.txt",stringsAsFactors = F)
sum(probes[,1] == origin_matrix$V1)
rownames(origin_matrix) <- origin_matrix$V1
origin_matrix$V1 <- NULL
##following part calculate for each probe, how many missing values-------------------------------
na_matrix <- is.na(as.matrix(origin_matrix))
na_num <- apply(na_matrix,1,sum)
na_summary <- data.frame(Probe = probes[,1],NA.num = na_num)
delete.probes.1 <- as.character(na_summary[na_summary$NA.num > 0,]$Probe)
##probes need to be deleted based on literatures and location-----------------------------------
cross_reactive <- read.csv("48639-non-specific-probes-Illumina450k_1st.csv",header = T,stringsAsFactors = F)
genomic_factor_probes <- read.csv("12864_2013_7006_MOESM2_ESM.csv",header = T,stringsAsFactors = F)
genomic_deleted <- genomic_factor_probes[genomic_factor_probes$Flag.discard.keep. == "discard",]
delete.probes.2 <- unique(c(cross_reactive$TargetID,genomic_deleted$probe))
probe_info <- fread("GPL13534-11288.txt",sep = "\t",header = T,skip = 37)
XY <- probe_info[probe_info$CHR %in% c("Y","X","")] #total 11713 probes
all.delete.probes <-unique(c(delete.probes.1,delete.probes.2,XY$ID))
#filter beta matrix------------------------------------------------------------------------------
final_probes <- rownames(origin_matrix)[-which(rownames(origin_matrix) %in% all.delete.probes)]
final_matrix <- origin_matrix[-which(rownames(origin_matrix) %in% all.delete.probes),]

##----------------------------------------------------------------------------------------------
final_info <- fread("BLCA_final_info.txt",sep = "\t",header = T)
setkey(final_info,person)
##calculate average beta
final_matrix <- subset(final_matrix,select = final_info$person)
sum(colnames(final_matrix) == final_info$person)
normals <- final_info[final_info$metast_info == "normal"]$person
locs <- final_info[final_info$metast_info == "no_metast"]$person
metasts <- final_info[final_info$metast_info == "metast"]$person
normal_matrix <- subset(final_matrix,select=normals)
sum(colnames(normal_matrix)==normals)
loc_matrix <- subset(final_matrix,select=locs)
sum(colnames(loc_matrix)==locs)
metast_matrix <- subset(final_matrix,select=metasts)
sum(colnames(metast_matrix)==metasts)
normal_mean <- apply(normal_matrix,1,mean)
loc_mean <- apply(loc_matrix,1,mean)
metast_mean <- apply(metast_matrix,1,mean)
```

```
mean_summary <- data.frame(probes = final_probes,normal = normal_mean,localized = loc_mean,metast = metast_mean)
##multi comparison by limma
data <- as.matrix(subset(final_matrix,select = final_info$person))
rownames(data) <- final_probes
sum(colnames(data) == final_info$person)
M_data <- log2(data / (1 - data))
Group <- factor(final_info$metast_info, levels=c("normal","no_metast","metast"))
design <- model.matrix(~0+Group)
colnames(design) <- c("normal","no_metast","metast")
fit <- lmFit(M_data, design)
contrast.matrix <- makeContrasts(no_metast-normal, metast-normal, levels=design)
fit.1 <- contrasts.fit(fit, contrast.matrix)
fit.1 <- eBayes(fit.1)
result_loc_nor <- topTable(fit.1,coef = 1,number = 485577,adjust="BH")
result_met_nor <- topTable(fit.1,coef = 2,number = 485577,adjust="BH")
result_loc_nor <- result_loc_nor[final_probes,]
result_met_nor <- result_met_nor[final_probes,]
sum(final_probes == mean_summary$probes)
final_result <- data.frame(Probe = final_probes,Loc.Nor.FDR = result_loc_nor$adj.P.Val,
                           Met.Nor.FDR = result_met_nor$adj.P.Val,normal.mean = normal_mean,
                           localized.mean = loc_mean,metast.mean = metast_mean)
final_result$beta.delta <- final_result$localized.mean - final_result$normal.mean
fwrite(final_result,file = "BLCA_result_limma.txt",sep = "\t",quote = F,col.names = T,row.names = F)
```

pan_cancer_analysis.R

```
#Script summarize for each site and prepare for next step (pan-cancer biomarker selection)
library(data.table)

##read in all limma result files and get a list of significant site for each cancer type----------
BLCA <- fread("BLCA_result_limma.txt",header = T,sep = "\t")
BLCA_sign_up <- BLCA[BLCA$Loc.Nor.FDR < 0.05 & BLCA$Met.Nor.FDR < 0.05 &
                     BLCA$beta.delta > 0 & BLCA$metast.mean - BLCA$normal.mean > 0]
BLCA_sign_down <- BLCA[BLCA$Loc.Nor.FDR < 0.05 & BLCA$Met.Nor.FDR < 0.05 &
                       BLCA$beta.delta < 0 & BLCA$metast.mean - BLCA$normal.mean < 0]

DBRCA <- fread("DBRCA_result_limma.txt",header = T,sep = "\t")
DBRCA_sign_up <- DBRCA[DBRCA$Loc.Nor.FDR < 0.05 & DBRCA$Met.Nor.FDR < 0.05 &
                       DBRCA$beta.delta > 0 & DBRCA$metast.mean - DBRCA$normal.mean > 0]
DBRCA_sign_down <- DBRCA[DBRCA$Loc.Nor.FDR < 0.05 & DBRCA$Met.Nor.FDR < 0.05 &
                         DBRCA$beta.delta < 0 & DBRCA$metast.mean - DBRCA$normal.mean < 0]

LBRCA <- fread("LBRCA_result_limma.txt",header = T,sep = "\t")
LBRCA_sign_up <- LBRCA[LBRCA$Loc.Nor.FDR < 0.05 & LBRCA$Met.Nor.FDR < 0.05 &
                       LBRCA$beta.delta > 0 & LBRCA$metast.mean - LBRCA$normal.mean > 0]
LBRCA_sign_down <- LBRCA[LBRCA$Loc.Nor.FDR < 0.05 & LBRCA$Met.Nor.FDR < 0.05 &
                         LBRCA$beta.delta < 0 & LBRCA$metast.mean - LBRCA$normal.mean < 0]

COAD <- fread("COAD_result_limma.txt",header = T,sep = "\t")
COAD_sign_up <- COAD[COAD$Loc.Nor.FDR < 0.05 & COAD$Met.Nor.FDR < 0.05 &
                     COAD$beta.delta > 0 & COAD$metast.mean - COAD$normal.mean > 0]
COAD_sign_down <- COAD[COAD$Loc.Nor.FDR < 0.05 & COAD$Met.Nor.FDR < 0.05 &
                       COAD$beta.delta < 0 & COAD$metast.mean - COAD$normal.mean < 0]

ESCA <- fread("ESCA_result_limma.txt",header = T,sep = "\t")
ESCA_sign_up <- ESCA[ESCA$Loc.Nor.FDR < 0.05 & ESCA$Met.Nor.FDR < 0.05 &
                     ESCA$beta.delta > 0 & ESCA$metast.mean - ESCA$normal.mean > 0]
ESCA_sign_down <- ESCA[ESCA$Loc.Nor.FDR < 0.05 & ESCA$Met.Nor.FDR < 0.05 &
                       ESCA$beta.delta < 0 & ESCA$metast.mean - ESCA$normal.mean < 0]

HNSC <- fread("HNSC_result_limma.txt",header = T,sep = "\t")
HNSC_sign_up <- HNSC[HNSC$Loc.Nor.FDR < 0.05 & HNSC$Met.Nor.FDR < 0.05 &
                     HNSC$beta.delta > 0 & HNSC$metast.mean - HNSC$normal.mean > 0]
HNSC_sign_down <- HNSC[HNSC$Loc.Nor.FDR < 0.05 & HNSC$Met.Nor.FDR < 0.05 &
                       HNSC$beta.delta < 0 & HNSC$metast.mean - HNSC$normal.mean < 0]

KIRC <- fread("KIRC_result_limma.txt",header = T,sep = "\t")
KIRC_sign_up <- KIRC[KIRC$Loc.Nor.FDR < 0.05 & KIRC$Met.Nor.FDR < 0.05 &
                     KIRC$beta.delta > 0 & KIRC$metast.mean - KIRC$normal.mean > 0]
KIRC_sign_down <- KIRC[KIRC$Loc.Nor.FDR < 0.05 & KIRC$Met.Nor.FDR < 0.05 &
                       KIRC$beta.delta < 0 & KIRC$metast.mean - KIRC$normal.mean < 0]
```

```
KIRP <- fread("KIRP_result_limma.txt",header = T,sep = "\t")
KIRP_sign_up <- KIRP[KIRP$Loc.Nor.FDR < 0.05 & KIRP$Met.Nor.FDR < 0.05 &
                     KIRP$beta.delta > 0 & KIRP$metast.mean - KIRP$normal.mean > 0]
KIRP_sign_down <- KIRP[KIRP$Loc.Nor.FDR < 0.05 & KIRP$Met.Nor.FDR < 0.05 &
                       KIRP$beta.delta < 0 & KIRP$metast.mean - KIRP$normal.mean < 0]

LIHC <- fread("LIHC_result_limma.txt",header = T,sep = "\t")
LIHC_sign_up <- LIHC[LIHC$Loc.Nor.FDR < 0.05 & LIHC$Met.Nor.FDR < 0.05 &
                     LIHC$beta.delta > 0 & LIHC$metast.mean - LIHC$normal.mean > 0]
LIHC_sign_down <- LIHC[LIHC$Loc.Nor.FDR < 0.05 & LIHC$Met.Nor.FDR < 0.05 &
                       LIHC$beta.delta < 0 & LIHC$metast.mean - LIHC$normal.mean < 0]

LUAD <- fread("LUAD_result_limma.txt",header = T,sep = "\t")
LUAD_sign_up <- LUAD[LUAD$Loc.Nor.FDR < 0.05 & LUAD$Met.Nor.FDR < 0.05 &
                     LUAD$beta.delta > 0 & LUAD$metast.mean - LUAD$normal.mean > 0]
LUAD_sign_down <- LUAD[LUAD$Loc.Nor.FDR < 0.05 & LUAD$Met.Nor.FDR < 0.05 &
                       LUAD$beta.delta < 0 & LUAD$metast.mean - LUAD$normal.mean < 0]

LUSC <- fread("LUSC_result_limma.txt",header = T,sep = "\t")
LUSC_sign_up <- LUSC[LUSC$Loc.Nor.FDR < 0.05 & LUSC$Met.Nor.FDR < 0.05 &
                     LUSC$beta.delta > 0 & LUSC$metast.mean - LUSC$normal.mean > 0]
LUSC_sign_down <- LUSC[LUSC$Loc.Nor.FDR < 0.05 & LUSC$Met.Nor.FDR < 0.05 &
                       LUSC$beta.delta < 0 & LUSC$metast.mean - LUSC$normal.mean < 0]

PAAD <- fread("PAAD_result_limma.txt",header = T,sep = "\t")
PAAD_sign_up <- PAAD[PAAD$Loc.Nor.FDR < 0.05 & PAAD$Met.Nor.FDR < 0.05 &
                     PAAD$beta.delta > 0 & PAAD$metast.mean - PAAD$normal.mean > 0]
PAAD_sign_down <- PAAD[PAAD$Loc.Nor.FDR < 0.05 & PAAD$Met.Nor.FDR < 0.05 &
                       PAAD$beta.delta < 0 & PAAD$metast.mean - PAAD$normal.mean < 0]

PRAD <- fread("PRAD_result_limma.txt",header = T,sep = "\t")
PRAD_sign_up <- PRAD[PRAD$Loc.Nor.FDR < 0.05 & PRAD$Met.Nor.FDR < 0.05 &
                     PRAD$beta.delta > 0 & PRAD$metast.mean - PRAD$normal.mean > 0]
PRAD_sign_down <- PRAD[PRAD$Loc.Nor.FDR < 0.05 & PRAD$Met.Nor.FDR < 0.05 &
                       PRAD$beta.delta < 0 & PRAD$metast.mean - PRAD$normal.mean < 0]

READ <- fread("READ_result_limma.txt",header = T,sep = "\t")
READ_sign_up <- READ[READ$Loc.Nor.FDR < 0.05 & READ$Met.Nor.FDR < 0.05 &
                     READ$beta.delta > 0 & READ$metast.mean - READ$normal.mean > 0]
READ_sign_down <- READ[READ$Loc.Nor.FDR < 0.05 & READ$Met.Nor.FDR < 0.05 &
                       READ$beta.delta < 0 & READ$metast.mean - READ$normal.mean < 0]

FTHCA <- fread("FTHCA_result_limma.txt",header = T,sep = "\t")
FTHCA_sign_up <- FTHCA[FTHCA$Loc.Nor.FDR < 0.05 & FTHCA$Met.Nor.FDR < 0.05 &
                       FTHCA$beta.delta > 0 & FTHCA$metast.mean - FTHCA$normal.mean > 0]
FTHCA_sign_down <- FTHCA[FTHCA$Loc.Nor.FDR < 0.05 & FTHCA$Met.Nor.FDR < 0.05 &
                         FTHCA$beta.delta < 0 & FTHCA$metast.mean - FTHCA$normal.mean < 0]

PTHCA <- fread("PTHCA_result_limma.txt",header = T,sep = "\t")
```

```
PTHCA_sign_up <- PTHCA[PTHCA$Loc.Nor.FDR < 0.05 & PTHCA$Met.Nor.FDR < 0.05 &
                                        PTHCA$beta.delta > 0 & PTHCA$metast.mean - PTHCA$normal.mean > 0]
PTHCA_sign_down <- PTHCA[PTHCA$Loc.Nor.FDR < 0.05 & PTHCA$Met.Nor.FDR < 0.05 &
                                          PTHCA$beta.delta < 0 & PTHCA$metast.mean - PTHCA$normal.mean < 0]

#summarize for each site
all.sign.up.probes <- c(BLCA_sign_up$Probe,DBRCA_sign_up$Probe,LBRCA_sign_up$Probe,COAD_sign_up$Probe,
                                    ESCA_sign_up$Probe,HNSC_sign_up$Probe,KIRC_sign_up$Probe,KIRP_sign_up$Probe,
                                    LIHC_sign_up$Probe,LUAD_sign_up$Probe,LUSC_sign_up$Probe,PAAD_sign_up$Probe,
                                    PRAD_sign_up$Probe,READ_sign_up$Probe,FTHCA_sign_up$Probe,PTHCA_sign_up$Probe)

all.sign.down.probes <- c(BLCA_sign_down$Probe,DBRCA_sign_down$Probe,LBRCA_sign_down$Probe,
                                      COAD_sign_down$Probe,ESCA_sign_down$Probe,HNSC_sign_down$Probe,
                                      KIRC_sign_down$Probe,KIRP_sign_down$Probe,LIHC_sign_down$Probe,
                                      LUAD_sign_down$Probe,LUSC_sign_down$Probe,PAAD_sign_down$Probe,
                                      PRAD_sign_down$Probe,READ_sign_down$Probe,FTHCA_sign_down$Probe,
                                      PTHCA_sign_down$Probe)
up_sum <- as.data.frame(table(all.sign.up.probes))
down_sum <- as.data.frame(table(all.sign.down.probes))

write.table(up_sum,"up_methylated_probe_sum.txt",sep = "\t",quote = F,row.names = F,col.names = T)
write.table(down_sum,"down_methylated_probe_sum.txt",sep = "\t",quote = F,row.names = F,col.names = T)
```

TCGA_PRAD_Gleason_process.R

#Script to apply comparison analysis between normal tissues and multiple Gleason score tumors by limma

```
library(data.table)
library(limma)

##original files---------------------------------------------------------------------------------
origin_matrix <- fread("PRAD/beta_matrix_final",header = T,sep = "\t")
probes <- read.table("probes.txt",stringsAsFactors = F)
sum(probes[,1] == origin_matrix$V1)
rownames(origin_matrix) <- origin_matrix$V1
origin_matrix$V1 <- NULL
##following part calculate for each probe, how many missing values-------------------------------
na_matrix <- is.na(as.matrix(origin_matrix))
na_num <- apply(na_matrix,1,sum)
na_summary <- data.frame(Probe = probes[,1],NA.num = na_num)
delete.probes.1 <- as.character(na_summary[na_summary$NA.num > 0,]$Probe)
##probes need to be deleted based on literatures and location------------------------------------
cross_reactive <- read.csv("48639-non-specific-probes-Illumina450k_1st.csv",header = T,stringsAsFactors = F)
genomic_factor_probes <- read.csv("12864_2013_7006_MOESM2_ESM.csv",header = T,stringsAsFactors = F)
genomic_deleted <- genomic_factor_probes[genomic_factor_probes$Flag.discard.keep. == "discard",]
delete.probes.2 <- unique(c(cross_reactive$TargetID,genomic_deleted$probe))
probe_info <- fread("GPL13534-11288.txt",sep = "\t",header = T,skip = 37)
XY <- probe_info[probe_info$CHR %in% c("Y","X","")] #total 11713 probes
all.delete.probes <-unique(c(delete.probes.1,delete.probes.2,XY$ID))

#read the clinical information and classify based on Gleason scores----------------------------
clinical_info <- fread("PRAD_clinical.txt",sep = "\t",header = T)
clinical_info <- subset(clinical_info,select = c("CASE_ID","AGE","RACE","GLEASON_PATTERN_PRIMARY","GLEASON_PATTERN_SECONDARY","GLEASON_PATTERN_TERTIARY","GLEASON_SCORE","PSA_MOST_RECENT_RESULTS"))
person_list <- as.character()
for (i in 1:nrow(clinical_info)) {
  total <- clinical_info$CASE_ID[i]
  split_info <- unlist(strsplit(x = total,split = '-',fixed = T))
  person <- paste(paste(split_info[4],'A',sep = ''),split_info[3],sep = '.')
  person_list <- c(person_list,person)
}
clinical_info$person <- person_list
clinical_info$new.GLEASON[clinical_info$GLEASON_SCORE == 6] <- "Gleason.1"
clinical_info$new.GLEASON[clinical_info$GLEASON_PATTERN_PRIMARY == 3 & clinical_info$GLEASON_PATTERN_SECONDARY == 4] <- "Gleason.2"
clinical_info$new.GLEASON[clinical_info$GLEASON_PATTERN_PRIMARY == 4 & clinical_info$GLEASON_PATTERN_SECONDARY == 3] <- "Gleason.3"
clinical_info$new.GLEASON[clinical_info$GLEASON_SCORE == 8] <- "Gleason.4"
clinical_info$new.GLEASON[clinical_info$GLEASON_SCORE %in% 9:10] <- "Gleason.5"

PRAD.final_info <- fread("PRAD_final_info.txt",sep = "\t",header = T)
```

```
PRAD.normals <- PRAD.final_info[PRAD.final_info$metast_info == "normal"]$person
PRAD.tumors <- PRAD.final_info[PRAD.final_info$metast_info != "normal"]$person
clinical_info <- clinical_info[clinical_info$person %in% colnames(origin_matrix)]
barplot(table(clinical_info$GLEASON_SCORE))
table(clinical_info$new.GLEASON)
barplot(table(clinical_info$new.GLEASON))
fwrite(clinical_info,"PRAD_gleason_info.txt",sep = "\t",quote = F,row.names = F,col.names = T)

#compare all five Gleason groups by limma---------------------------------------------------
final_probes <- rownames(origin_matrix)[-which(rownames(origin_matrix) %in% all.delete.probes)]
final_matrix <- origin_matrix[-which(rownames(origin_matrix) %in% all.delete.probes),]
final_matrix <- subset(final_matrix,select = clinical_info$person)
sum(colnames(final_matrix) == clinical_info$person)
##multi comparison - use the five Gleason groups and compare all groups
data <- as.matrix(final_matrix)
rownames(data) <- final_probes
sum(colnames(data) == clinical_info$person)
M_data <- log2(data / (1 - data))
Group <- factor(clinical_info$new.GLEASON, levels=c("Gleason.1","Gleason.2","Gleason.3","Gleason.4","Gleason.5"))
design <- model.matrix(~0+Group)
colnames(design) <- c("Gleason.1","Gleason.2","Gleason.3","Gleason.4","Gleason.5")
fit <- lmFit(M_data, design)
contrast.matrix <- makeContrasts(Gleason.1-Gleason.2, Gleason.2-Gleason.3,Gleason.3-Gleason.4,
                                 Gleason.4-Gleason.5,levels=design)
fit.1 <- contrasts.fit(fit, contrast.matrix)
fit.1 <- eBayes(fit.1)
result_1_2 <- topTable(fit.1,coef = 1,number = 485577,adjust="BH")
result_2_3 <- topTable(fit.1,coef = 2,number = 485577,adjust="BH")
result_3_4 <- topTable(fit.1,coef = 3,number = 485577,adjust="BH")
result_4_5 <- topTable(fit.1,coef = 4,number = 485577,adjust="BH")
result_1_2 <- result_1_2[final_probes,]
result_2_3 <- result_2_3[final_probes,]
result_3_4 <- result_3_4[final_probes,]
result_4_5 <- result_4_5[final_probes,]

final_result <- data.table(Probe = final_probes,FDR.1.2 = result_1_2$adj.P.Val,FDR.2.3 = result_2_3$adj.P.Val,
                           FDR.3.4 = result_3_4$adj.P.Val, FDR.4.5 = result_4_5$adj.P.Val)
fwrite(final_result,"PRAD_Gleason_5_groups_diff.txt",quote = F,sep = "\t",row.names = F,col.names = T)



#compare only three Gleason groups by limma---------------------------------------------------
clinical_info$three.GLEASON <- ifelse(clinical_info$new.GLEASON %in% c("Gleason.1","Gleason.2"),"GL12",
                                      ifelse(clinical_info$new.GLEASON %in% c("Gleason.3","Gleason.4"),
                                             "GL34","GL5"))
final_info <- data.table(Person = c(PRAD.normals,clinical_info$person),GL =
c(rep("normal",length(PRAD.normals)),clinical_info$three.GLEASON))

final_probes <- rownames(origin_matrix)[-which(rownames(origin_matrix) %in% all.delete.probes)]
final_matrix <- origin_matrix[-which(rownames(origin_matrix) %in% all.delete.probes),]
```

```
final_matrix <- subset(final_matrix,select = final_info$Person)
sum(colnames(final_matrix) == final_info$Person)

data <- as.matrix(final_matrix)
rownames(data) <- final_probes
sum(colnames(data) == final_info$Person)
M_data <- log2(data / (1 - data))
Group <- factor(final_info$GL, levels=c("normal","GL12","GL34","GL5"))
design <- model.matrix(~0+Group)
colnames(design) <- c("normal","GL12","GL34","GL5")
fit <- lmFit(M_data, design)
contrast.matrix <- makeContrasts(normal-GL12, normal-GL34, normal-GL5,
                                 GL12-GL34, GL12-GL5, GL34-GL5,levels=design)
fit.1 <- contrasts.fit(fit, contrast.matrix)
fit.1 <- eBayes(fit.1)
result_n_12 <- topTable(fit.1,coef = 1,number = 485577,adjust="BH")
result_n_34 <- topTable(fit.1,coef = 2,number = 485577,adjust="BH")
result_n_5 <- topTable(fit.1,coef = 3,number = 485577,adjust="BH")
result_12_34 <- topTable(fit.1,coef = 4,number = 485577,adjust="BH")
result_12_5 <- topTable(fit.1,coef = 5,number = 485577,adjust="BH")
result_34_5 <- topTable(fit.1,coef = 6,number = 485577,adjust="BH")
result_n_12 <- result_n_12[final_probes,]
result_n_34 <- result_n_34[final_probes,]
result_n_5 <- result_n_5[final_probes,]
result_12_34 <- result_12_34[final_probes,]
result_12_5 <- result_12_5[final_probes,]
result_34_5 <- result_34_5[final_probes,]

#calculate average beta values
normals <- final_info[final_info$GL == "normal"]$Person
GL12 <- final_info[final_info$GL == "GL12"]$Person
GL34 <- final_info[final_info$GL == "GL34"]$Person
GL5 <- final_info[final_info$GL == "GL5"]$Person
normal_matrix <- subset(final_matrix,select=normals)
sum(colnames(normal_matrix)==normals)
GL12_matrix <- subset(final_matrix,select=GL12)
sum(colnames(GL12_matrix)==GL12)
GL34_matrix <- subset(final_matrix,select=GL34)
sum(colnames(GL34_matrix)==GL34)
GL5_matrix <- subset(final_matrix,select=GL5)
sum(colnames(GL5_matrix)==GL5)
normal_mean <- apply(normal_matrix,1,mean)
GL12_mean <- apply(GL12_matrix,1,mean)
GL34_mean <- apply(GL34_matrix,1,mean)
GL5_mean <- apply(GL5_matrix,1,mean)

final_result <- data.table(Probe = final_probes,FDR.n.12 = result_n_12$adj.P.Val,
                           FDR.n.34 = result_n_34$adj.P.Val, FDR.n.5 = result_n_5$adj.P.Val,
                           FDR.12.34 = result_12_34$adj.P.Val, FDR.12.5 = result_12_5$adj.P.Val,
```

```
                                    FDR.34.5 = result_34_5$adj.P.Val, normal.mean = normal_mean,
                                    GL12.mean = GL12_mean, GL34.mean = GL34_mean, GL5.mean = GL5_mean)
fwrite(final_result,"PRAD_normal_3GL_groups_diff.txt",quote = F,sep = "\t",row.names = F,col.names = T)
```

TCGA_PRAD_nonPRAD_process.R

#Script to apply limma analysis between PRAD and other urinary samples

```
library(data.table)
library(limma)

##read and process original files--------------------------------------------------------------------------
BLCA.origin_matrix <- fread("BLCA/beta_matrix_final",header = T,sep = "\t")
KIRC.origin_matrix <- fread("KIRC/beta_matrix_final",header = T,sep = "\t")
KIRP.origin_matrix <- fread("KIRP/beta_matrix_final",header = T,sep = "\t")
PRAD.origin_matrix <- fread("PRAD/beta_matrix_final",header = T,sep = "\t")
setkey(BLCA.origin_matrix,V1)
setkey(KIRC.origin_matrix,V1)
setkey(KIRP.origin_matrix,V1)
setkey(PRAD.origin_matrix,V1)

BLCA.normals <- colnames(BLCA.origin_matrix)[grep('11A.',colnames(BLCA.origin_matrix))]
BLCA.tumors <- colnames(BLCA.origin_matrix)[grep('01A.',colnames(BLCA.origin_matrix))]
KIRC.normals <- colnames(KIRC.origin_matrix)[grep('11A.',colnames(KIRC.origin_matrix))]
KIRC.tumors <- colnames(KIRC.origin_matrix)[grep('01A.',colnames(KIRC.origin_matrix))]
KIRP.normals <- colnames(KIRP.origin_matrix)[grep('11A.',colnames(KIRP.origin_matrix))]
KIRP.tumors <- colnames(KIRP.origin_matrix)[grep('01A.',colnames(KIRP.origin_matrix))]
PRAD.normals <- colnames(PRAD.origin_matrix)[grep('11A.',colnames(PRAD.origin_matrix))]
PRAD.tumors <- colnames(PRAD.origin_matrix)[grep('01A.',colnames(PRAD.origin_matrix))]

BLCA.final_info <- data.table(person = c(BLCA.normals,BLCA.tumors),type = rep("others",(ncol(BLCA.origin_matrix)-1)))
KIRC.final_info <- data.table(person = c(KIRC.normals,KIRC.tumors),type = rep("others",(ncol(KIRC.origin_matrix)-1)))
KIRP.final_info <- data.table(person = c(KIRP.normals,KIRP.tumors),type = rep("others",(ncol(KIRP.origin_matrix)-1)))
PRAD.final_info <- data.table(person = c(PRAD.normals,PRAD.tumors),type = 
c(rep("others",length(PRAD.normals)),rep("PRAD",length(PRAD.tumors))))

cross_reactive <- read.csv("48639-non-specific-probes-Illumina450k_1st.csv",header = T,stringsAsFactors = F)
genomic_factor_probes <- read.csv("12864_2013_7006_MOESM2_ESM.csv",header = T,stringsAsFactors = F)
genomic_deleted <- genomic_factor_probes[genomic_factor_probes$Flag.discard.keep. == "discard",]
probe_info <- fread("GPL13534-11288.txt",sep = "\t",header = T,skip = 37)
XY <- probe_info[probe_info$CHR %in% c("Y","X","")] #total 11713 probes
all.delete.probes <- unique(c(cross_reactive$TargetID,genomic_deleted$probe,XY$ID))
final_probes <- PRAD.origin_matrix$V1[-which(PRAD.origin_matrix$V1 %in% all.delete.probes)]

BLCA.matrix <- BLCA.origin_matrix[final_probes]
KIRC.matrix <- KIRC.origin_matrix[final_probes]
KIRP.matrix <- KIRP.origin_matrix[final_probes]
PRAD.matrix <- PRAD.origin_matrix[final_probes]

BLCA.matrix <- subset(BLCA.matrix,select = BLCA.final_info$person)
KIRC.matrix <- subset(KIRC.matrix,select = KIRC.final_info$person)
KIRP.matrix <- subset(KIRP.matrix,select = KIRP.final_info$person)
PRAD.matrix <- subset(PRAD.matrix,select = PRAD.final_info$person)
```

```
sum(colnames(PRAD.matrix) == PRAD.final_info$person)

cat.final_info <- rbind(BLCA.final_info,KIRC.final_info,KIRP.final_info,PRAD.final_info)
cat.matrix <- cbind(BLCA.matrix,KIRC.matrix,KIRP.matrix,PRAD.matrix)
PRAD.tumor.id <- cat.final_info[cat.final_info$type == "PRAD"]$person
others.id <- cat.final_info[cat.final_info$type == "others"]$person
PRAD.tumor.matrix <- subset(cat.matrix,select=PRAD.tumor.id)
others.matrix <- subset(cat.matrix,select=others.id)
mean_na <- function(x){mean(x,na.rm = T)}
PRAD.tumor.mean <- apply(PRAD.tumor.matrix,1,mean_na)
others.mean <- apply(others.matrix,1,mean_na)

#apply comparison analysis between PRAD and other urinary samples------------------------------
data <- as.matrix(cat.matrix)
rownames(data) <- final_probes
sum(colnames(data) == cat.final_info$person)
M_data <- log2(data / (1 - data))
Group <- factor(cat.final_info$type, levels=c("PRAD","others"))
design <- model.matrix(~Group)
colnames(design) <- c("inter","verse")
fit <- lmFit(M_data, design)
fit <- eBayes(fit)
result_PRAD_others <- topTable(fit,coef = "verse",number = 485577,adjust="BH")
result_PRAD_others <- result_PRAD_others[final_probes,]
final_result <- data.table(Probes = final_probes,FDR = result_PRAD_others$adj.P.Val,
                                        PRAD.tumor.mean = PRAD.tumor.mean,others.mean = others.mean)
final_result <- na.omit(final_result)
final_result$delta.beta <- final_result$PRAD.tumor.mean - final_result$others.mean
fwrite(final_result,"PRAD_vs_urinary_result.txt",sep = "\t",quote = F,row.names = F,col.names = T)
```

LAR_n_5GL_bootstrap.R

#script to use LASSO Bootstrap method to further select sites with the potential to separate among normal and 5 Gleason groups

```
library(lars)
library(data.table)

##lar method on all PRAD tumor probes to select probes separate GL1 GL2 GL3
cluster_result <- fread("delete_corr0.8_probelist.txt",header = T,sep = "\t")
colnames(cluster_result) <- "Probe"
PRAD.final_info <- fread("PRAD/PRAD_final_info.txt",sep = "\t",header = T)
normals <- PRAD.final_info[PRAD.final_info$metast_info == "normal"]
PRAD.clinical_info <- fread("../02_biomarker_selection/PRAD_gleason_info.txt",sep = "\t",header = T)
PRAD.clinical_info$five.group.gl[PRAD.clinical_info$new.GLEASON == "Gleason.1"] <- 0
PRAD.clinical_info$five.group.gl[PRAD.clinical_info$new.GLEASON == "Gleason.2"] <- 1
PRAD.clinical_info$five.group.gl[PRAD.clinical_info$new.GLEASON == "Gleason.3"] <- 2
PRAD.clinical_info$five.group.gl[PRAD.clinical_info$new.GLEASON == "Gleason.4"] <- 3
PRAD.clinical_info$five.group.gl[PRAD.clinical_info$new.GLEASON == "Gleason.5"] <- 4

final_clinical_info <- data.table(person = c(normals$person,PRAD.clinical_info$person),
                                  GL = c(rep(-1,nrow(normals)),PRAD.clinical_info$five.group.gl))
origin_matrix <- fread("PRAD/beta_matrix_final",header = T,sep = "\t")
setkey(origin_matrix,V1)
final_matrix <- origin_matrix[cluster_result$Probe]
sum(final_matrix$V1 == cluster_result$Probe)
final_matrix <- subset(final_matrix,select = final_clinical_info$person)
sum(colnames(final_matrix) == final_clinical_info$person)
final_matrix_M <- log2(final_matrix / (1 - final_matrix))

#start bootstrap step
data <- as.data.table(t(as.matrix(final_matrix_M)))
colnames(data) <- cluster_result$Probe
data$GL <- factor(final_clinical_info$GL,levels = c(-1,0,1,2,3,4))
seed_list <- fread("model_construct/seeds_list.txt")
seeds1 <- seed_list$seeds1
sum_final_lasso <- data.table(c(cluster_result$Probe,"Seed"))
for (i in seeds1[1:1000]) {
  set.seed(i)
  train <- sample(nrow(data), nrow(data),replace = TRUE)
  df.train <- data[train,]
  df.train$GL <- NULL
  df.train <- apply(df.train,2,as.numeric)
  la <- lars(as.matrix(df.train),final_clinical_info$GL[train],type="lar")
  la_result <- data.table(Df = la$df,CP = la$Cp)
  num <- la_result[la_result$CP == min(la_result$CP)]$Df
  coef <- coef(la, s=num, mode="step")
  final_lasso_result.gl <- data.table(Probe = names(coef),Coef = as.numeric(coef))
  setkey(final_lasso_result.gl,Probe)
```

```
    final_lasso_result.gl <- final_lasso_result.gl[cluster_result$Probe]
    sum_final_lasso <- cbind(sum_final_lasso,c(final_lasso_result.gl$Coef,i))
    print(i)
}

zero <- function(x){sum(x==0)}
zero_num <- apply(sum_final_lasso[,-1],1,zero)
length(apply(sum_final_lasso,1,zero))
sum_final_lasso$zeros <- zero_num
fwrite(sum_final_lasso,"lasso1000_n_5gl.txt",quote = F,sep = "\t",row.names = F,col.names = T)
```

glm_gls_final.R

```
#script to fit generalized linear models by 70% data and validate by the rest 30% data
#Three models: 8-sites+PSA+age, 8-sites, PSA+age
#compare GL6 vs. GL3+4 and GL6 vs. GL4+3,8-10
library(AUC)

seed_list <- fread("seeds_list.txt")
seeds1 <- seed_list$seeds1

lasso_result <- fread("../lasso1000_n_5gl.txt",header = T,sep = "\t")
selected_probes <- lasso_result[lasso_result$zeros < 10]$V1
selected_probes <- selected_probes[-length(selected_probes)] # the last item is "Seed"

PRAD.final_info <- fread("PRAD/PRAD_final_info.txt",sep = "\t",header = T)
get_person <- function(x){unlist(strsplit(x,split = '.',fixed = T))[2]}
PRAD.final_info$person.id <- apply(as.matrix(PRAD.final_info$person),1,get_person)
PRAD.clinical_info <- fread("PRAD_gleason_info.txt",sep = "\t",header = T)
PRAD.clinical_info$person.id <- apply(as.matrix(PRAD.clinical_info$person),1,get_person)
setkey(PRAD.clinical_info,person.id)
final_clinical_info <- data.table(person = PRAD.clinical_info$person,
                                  Age = PRAD.clinical_info$AGE,
                                  PSA = PRAD.clinical_info$PSA_MOST_RECENT_RESULTS,
                                  new.GL = PRAD.clinical_info$new.GLEASON)
# final_clinical_info <- final_clinical_info[final_clinical_info$new.GL %in% c("Gleason.1","Gleason.2")]
final_clinical_info <- final_clinical_info[final_clinical_info$new.GL != "Gleason.2"]

origin_matrix <- fread("PRAD/beta_matrix_final",header = T,sep = "\t")
setkey(origin_matrix,V1)
final_matrix <- origin_matrix[selected_probes]
sum(final_matrix$V1 == selected_probes)
final_matrix <- subset(final_matrix,select = final_clinical_info$person)
sum(colnames(final_matrix) == final_clinical_info$person)
final_matrix_M <- log2(final_matrix / (1 - final_matrix))


#2 GL groups - with/without info of age and PSA - compare GL6 and GL3+4/GL4+3..10
a.acc <- numeric()
gl1.acc <- numeric()
gl23.acc <- numeric()
aucs <- numeric()
df <- as.data.table(t(as.matrix(final_matrix_M)))
# df$age <- final_clinical_info$Age
# df$PSA <- final_clinical_info$PSA
df$class <- factor(final_clinical_info$new.GL)
df$class <- ifelse(df$class == "Gleason.1","GL1","GL23")
df <- na.omit(df)
# colnames(df) <- c(selected_probes,"age","PSA","class")
```

```
colnames(df) <- c(selected_probes,"class")
for (i in 1:1000) {
  set.seed(seeds1[i])
  train <- sample(nrow(df), 0.7*nrow(df))
  df.train <- df[train,]
  df.validate <- df[-train,]
  fit.logit <- glm(as.factor(class)~., data=df.train, family=binomial(),maxit = 25)
  prob <- predict(fit.logit, df.validate, type="response")
  logit.pred <- factor(prob > .5, levels=c(FALSE, TRUE), labels=c("GL1","GL23"))
  pref.table <- table(df.validate$class, logit.pred, dnn=c("Actual", "Predicted"))
  overall_acc <- (pref.table[1,1]+pref.table[2,2])/sum(pref.table)
  GL1_acc <- pref.table[1,1]/sum(pref.table[1,])
  GL2_3_acc <- pref.table[2,2]/sum(pref.table[2,])
  a.acc <- c(a.acc,overall_acc)
  gl1.acc <- c(gl1.acc,GL1_acc)
  gl23.acc <- c(gl23.acc,GL2_3_acc)
  print(i)
  aucs <- c(aucs,auc(roc(prob,factor(df.validate$class,levels = c("GL1","GL23"),labels = c(0,1)))))
}
model_acc <- data.table(No = 1:1000,Overall.accuracy = a.acc,
                        GL1.accuracy = gl1.acc,GL23.accuracy=gl23.acc,
                        AUC = aucs)
boxplot(model_acc[,-1])
# fwrite(model_acc,"glm_2gl_8_with_1vs2.txt",quote = F,sep = "\t",row.names = F,col.names = T)
# fwrite(model_acc,"glm_2gl_8_without_1vs2.txt",quote = F,sep = "\t",row.names = F,col.names = T)
# fwrite(model_acc,"glm_2gl_8_with_1vs3-5.txt",quote = F,sep = "\t",row.names = F,col.names = T)
fwrite(model_acc,"glm_2gl_8_without_1vs3-5.txt",quote = F,sep = "\t",row.names = F,col.names = T)


#2 GL groups - only info of age or PSA - compare GL6 and GL3+4/GL4+3..10
a.acc <- numeric()
gl1.acc <- numeric()
gl23.acc <- numeric()
aucs <- numeric()
df <- as.data.table(t(as.matrix(final_matrix_M)))
df$age <- final_clinical_info$Age
df$PSA <- final_clinical_info$PSA
df$class <- factor(final_clinical_info$new.GL)
df$class <- ifelse(df$class == "Gleason.1","GL1","GL23")
df <- na.omit(df)
colnames(df) <- c(selected_probes,"age","PSA","class")
for (i in 1:1000) {
  set.seed(seeds1[i])
  train <- sample(nrow(df), 0.7*nrow(df))
  df.train <- df[train,]
  df.validate <- df[-train,]
  fit.logit <- glm(as.factor(class)~PSA+age, data=df.train, family=binomial(),maxit = 25)
  prob <- predict(fit.logit, df.validate, type="response")
  logit.pred <- factor(prob > .5, levels=c(FALSE, TRUE), labels=c("GL1","GL23"))
```

```
    pref.table <- table(df.validate$class, logit.pred, dnn=c("Actual", "Predicted"))
    overall_acc <- (pref.table[1,1]+pref.table[2,2])/sum(pref.table)
    GL1_acc <- pref.table[1,1]/sum(pref.table[1,])
    GL2_3_acc <- pref.table[2,2]/sum(pref.table[2,])
    a.acc <- c(a.acc,overall_acc)
    gl1.acc <- c(gl1.acc,GL1_acc)
    gl23.acc <- c(gl23.acc,GL2_3_acc)
    print(i)
    aucs <- c(aucs,auc(roc(prob,factor(df.validate$class,levels = c("GL1","GL23"),labels = c(0,1)))))
}
model_acc <- data.table(No = 1:1000,Overall.accuracy = a.acc,
                                                GL1.accuracy = gl1.acc,GL23.accuracy=gl23.acc,
                                                AUC = aucs)
boxplot(model_acc[,-1])
# fwrite(model_acc,"glm_2gl_psa_age_1vs2.txt",quote = F,sep = "\t",row.names = F,col.names = T)
fwrite(model_acc,"glm_2gl_psa_age_1vs3-5.txt",quote = F,sep = "\t",row.names = F,col.names = T)
```

glm_n_gl_final.R

#script to validate model by 8 or less sites in separating normal and tumor, by GEO datasets

```
library(AUC)

lasso_result <- fread("../lasso1000_n_5gl.txt",header = T,sep = "\t")
selected_probes <- lasso_result[lasso_result$zeros < 10]$V1
selected_probes <- selected_probes[-length(selected_probes)] # the last item is "Seed"

PRAD.final_info <- fread("PRAD/PRAD_final_info.txt",sep = "\t",header = T)
get_person <- function(x){unlist(strsplit(x,split = '.',fixed = T))[2]}
PRAD.final_info$person.id <- apply(as.matrix(PRAD.final_info$person),1,get_person)
normals <- PRAD.final_info[PRAD.final_info$metast_info == "normal"]
PRAD.clinical_info <- fread("PRAD_gleason_info.txt",sep = "\t",header = T)
PRAD.clinical_info$person.id <- apply(as.matrix(PRAD.clinical_info$person),1,get_person)
setkey(PRAD.clinical_info,person.id)
PRAD.clinical_info.normal <- PRAD.clinical_info[normals$person.id]
final_clinical_info <- data.table(person = c(normals$person,PRAD.clinical_info$person),
                                  Age = c(PRAD.clinical_info.normal$AGE,PRAD.clinical_info$AGE),
                                  PSA                                                                   =
c(PRAD.clinical_info.normal$PSA_MOST_RECENT_RESULTS,PRAD.clinical_info$PSA_MOST_RECENT_RESULTS),
                                  GL = c(rep("normal",nrow(normals)),PRAD.clinical_info$new.GLEASON))

final_clinical_info$N.T <- ifelse(final_clinical_info$GL == "normal","normal","tumor")
origin_matrix <- fread("PRAD/beta_matrix_final",header = T,sep = "\t")
setkey(origin_matrix,V1)
final_matrix <- origin_matrix[selected_probes]
sum(final_matrix$V1 == selected_probes)
final_matrix <- subset(final_matrix,select = final_clinical_info$person)
sum(colnames(final_matrix) == final_clinical_info$person)
final_matrix_M <- log2(final_matrix / (1 - final_matrix))

##test model to separate normal and tumor samples -- GSE47915
GSE47915_matrix <- fread("GSE47915/GSE47915_beta_matrix.txt",header = T,sep = "\t")
setkey(GSE47915_matrix,ID_REF)
GSE47915_matrix <- GSE47915_matrix[selected_probes]
GSE47915_matrix$ID_REF <- NULL
GSE47915_matrix_M <- log2(GSE47915_matrix / (1 - GSE47915_matrix))
GSE47915_info <- c("normal","tumor","tumor","tumor","tumor","normal","normal","normal")

df <- as.data.table(t(as.matrix(final_matrix_M)))
df$class <- factor(final_clinical_info$N.T,levels = c("normal","tumor"))
colnames(df) <- c(selected_probes,"class")
df.train <- df
table(df.train$class)
df.validate <- as.data.table(t(as.matrix(GSE47915_matrix_M)))
df.validate$class <- factor(GSE47915_info,levels = c("normal","tumor"))
colnames(df.validate) <- c(selected_probes,"class")
```

```
fit.logit <- glm(as.factor(class)~., data=df.train, family=binomial(),maxit = 25)
summary(fit.logit)
logit.fit.reduced <- step(fit.logit)
summary(logit.fit.reduced)
prob <- predict(logit.fit.reduced, df.validate, type="response")
logit.pred <- factor(prob > .5, levels=c(FALSE, TRUE), labels=c("normal","tumor"))
logit.perf <- table(df.validate$class, logit.pred, dnn=c("Actual", "Predicted"))
logit.perf
roc_result1 <- roc(prob,factor(df.validate$class,levels = c("normal","tumor"),labels = c(0,1)))
plot(roc_result1,col="black")
auc(roc_result1) # 0.875

##test model to separate normal and tumor samples -- GSE112047
GSE112047_matrix <- fread("GSE112047/GSE112047_beta_matrix.txt",header = T,sep = "\t")
setkey(GSE112047_matrix,ID_REF)
GSE112047_matrix <- GSE112047_matrix[selected_probes]
GSE112047_matrix$ID_REF <- NULL
GSE112047_matrix <- as.matrix(GSE112047_matrix)
GSE112047_matrix <- apply(GSE112047_matrix,2,as.numeric)
GSE112047_matrix_M <- log2(GSE112047_matrix / (1 - GSE112047_matrix))

GSE112047_info <- fread("GSE112047/GSE112047_metadata.csv",sep = ",",header = T)
sum(colnames(GSE112047_matrix_M) == GSE112047_info$ID)
GSE112047_info$class <- ifelse(GSE112047_info$group == "N","normal","tumor")
df <- as.data.table(t(as.matrix(final_matrix_M)))
df$class <- factor(final_clinical_info$N.T,levels = c("normal","tumor"))
colnames(df) <- c(selected_probes,"class")
df.train <- df
table(df.train$class)
df.validate <- as.data.table(t(as.matrix(GSE112047_matrix_M)))
df.validate$class <- factor(GSE112047_info$class,levels = c("normal","tumor"))
colnames(df.validate) <- c(selected_probes,"class")
fit.logit <- glm(as.factor(class)~., data=df.train, family=binomial(),maxit = 25)
summary(fit.logit)
logit.fit.reduced <- step(fit.logit)
summary(logit.fit.reduced)
prob <- predict(logit.fit.reduced, df.validate, type="response")
logit.pred <- factor(prob > .5, levels=c(FALSE, TRUE), labels=c("normal","tumor"))
logit.perf <- table(df.validate$class, logit.pred, dnn=c("Actual", "Predicted"))
logit.perf
roc_result2 <- roc(prob,factor(df.validate$class,levels = c("normal","tumor"),labels = c(0,1)))
plot(roc_result2,col="black")
auc(roc_result2) # 0.9153226

##test model to separate normal and tumor samples -- GSE76938
GSE76938_matrix <- fread("GSE76938/GSE76938_beta_matrix.txt",header = T,sep = "\t")
setkey(GSE76938_matrix,ID_REF)
GSE76938_matrix <- GSE76938_matrix[selected_probes]
GSE76938_info <- fread("GSE76938/GSE76938_metadata.csv",sep = ",",header = T)
```

```
setkey(GSE76938_info,ID)
final_GSE76938_ID    <- GSE76938_info$ID
GSE76938_info <- GSE76938_info[final_GSE76938_ID]
GSE76938_matrix$ID_REF <- NULL
GSE76938_matrix <- subset(GSE76938_matrix,select = final_GSE76938_ID)
GSE76938_matrix <- as.matrix(GSE76938_matrix)
GSE76938_matrix <- apply(GSE76938_matrix,2,as.numeric)
GSE76938_matrix_M <- log2(GSE76938_matrix / (1 - GSE76938_matrix))
sum(colnames(GSE76938_matrix_M) == GSE76938_info$ID)

GSE76938_info$class <- ifelse(GSE76938_info$group == "N","normal","tumor")
df <- as.data.table(t(as.matrix(final_matrix_M)))
df$class <- factor(final_clinical_info$N.T,levels = c("normal","tumor"))
colnames(df) <- c(selected_probes,"class")
df.train <- df
table(df.train$class)
df.validate <- as.data.table(t(as.matrix(GSE76938_matrix_M)))
df.validate$class <- factor(GSE76938_info$class,levels = c("normal","tumor"))
colnames(df.validate) <- c(selected_probes,"class")
fit.logit <- glm(as.factor(class)~., data=df.train, family=binomial(),maxit = 25)
summary(fit.logit)
logit.fit.reduced <- step(fit.logit)
summary(logit.fit.reduced)
prob <- predict(logit.fit.reduced, df.validate, type="response")
logit.pred <- factor(prob > .5, levels=c(FALSE, TRUE), labels=c("normal","tumor"))
logit.perf <- table(df.validate$class, logit.pred, dnn=c("Actual", "Predicted"))
logit.perf
roc_result3 <- roc(prob,factor(df.validate$class,levels = c("normal","tumor"),labels = c(0,1)))
plot(roc_result3,col="black")
auc(roc_result3) # 0.8881279
```

glm_PRAD_nonPRAD.R

#script to validate model separating PRAD and nonPRAD samples by GEO dataset

```
library(AUC)

##lar method on all PRAD tumor probes to select probes separate PRAD and non-PRAD
BLCA.origin_matrix <- fread("BLCA/beta_matrix_final",header = T,sep = "\t")
KIRC.origin_matrix <- fread("KIRC/beta_matrix_final",header = T,sep = "\t")
KIRP.origin_matrix <- fread("KIRP/beta_matrix_final",header = T,sep = "\t")
PRAD.origin_matrix <- fread("PRAD/beta_matrix_final",header = T,sep = "\t")
setkey(BLCA.origin_matrix,V1)
setkey(KIRC.origin_matrix,V1)
setkey(KIRP.origin_matrix,V1)
setkey(PRAD.origin_matrix,V1)

BLCA.normals <- colnames(BLCA.origin_matrix)[grep('11A.',colnames(BLCA.origin_matrix))]
BLCA.tumors <- colnames(BLCA.origin_matrix)[grep('01A.',colnames(BLCA.origin_matrix))]
KIRC.normals <- colnames(KIRC.origin_matrix)[grep('11A.',colnames(KIRC.origin_matrix))]
KIRC.tumors <- colnames(KIRC.origin_matrix)[grep('01A.',colnames(KIRC.origin_matrix))]
KIRP.normals <- colnames(KIRP.origin_matrix)[grep('11A.',colnames(KIRP.origin_matrix))]
KIRP.tumors <- colnames(KIRP.origin_matrix)[grep('01A.',colnames(KIRP.origin_matrix))]
PRAD.normals <- colnames(PRAD.origin_matrix)[grep('11A.',colnames(PRAD.origin_matrix))]
PRAD.tumors <- colnames(PRAD.origin_matrix)[grep('01A.',colnames(PRAD.origin_matrix))]

BLCA.final_info <- data.table(person = c(BLCA.normals,BLCA.tumors),type = rep("others",(ncol(BLCA.origin_matrix)-1)))
KIRC.final_info <- data.table(person = c(KIRC.normals,KIRC.tumors),type = rep("others",(ncol(KIRC.origin_matrix)-1)))
KIRP.final_info <- data.table(person = c(KIRP.normals,KIRP.tumors),type = rep("others",(ncol(KIRP.origin_matrix)-1)))
PRAD.final_info <- data.table(person = c(PRAD.normals,PRAD.tumors),type = 
c(rep("others",length(PRAD.normals)),rep("PRAD.tumor",length(PRAD.tumors))))

lasso_result <- fread("lasso1000_n_5gl.txt",header = T,sep = "\t")
selected_probes <- lasso_result[lasso_result$zeros < 10]$V1
selected_probes <- selected_probes[-length(selected_probes)] # the last item is "Seed"

BLCA.matrix <- BLCA.origin_matrix[selected_probes]
KIRC.matrix <- KIRC.origin_matrix[selected_probes]
KIRP.matrix <- KIRP.origin_matrix[selected_probes]
PRAD.matrix <- PRAD.origin_matrix[selected_probes]

BLCA.matrix <- subset(BLCA.matrix,select = BLCA.final_info$person)
KIRC.matrix <- subset(KIRC.matrix,select = KIRC.final_info$person)
KIRP.matrix <- subset(KIRP.matrix,select = KIRP.final_info$person)
PRAD.matrix <- subset(PRAD.matrix,select = PRAD.final_info$person)

sum(is.na(as.matrix(cbind(BLCA.matrix,KIRC.matrix,KIRP.matrix,PRAD.matrix))))
sum(is.na(as.matrix(BLCA.matrix))) #2
sum(is.na(as.matrix(KIRC.matrix)))
sum(is.na(as.matrix(KIRP.matrix)))
```

```
sum(is.na(as.matrix(PRAD.matrix)))

#search for 2 NA and delete NA persons
delete.BLCA.matrix <- as.data.table(t(as.matrix(BLCA.matrix)))
rownames(delete.BLCA.matrix) <- BLCA.final_info$person
delete.BLCA.matrix$person <- BLCA.final_info$person
delete.BLCA.matrix <- na.omit(delete.BLCA.matrix)
sum(is.na(as.matrix(delete.BLCA.matrix)))
final_BLCA_person <- delete.BLCA.matrix$person
final.BLCA.matrix <- BLCA.origin_matrix[selected_probes]
sum(final.BLCA.matrix$V1 == selected_probes)
final.BLCA.final_info <- BLCA.final_info[BLCA.final_info$person %in% final_BLCA_person]
final.BLCA.matrix <- subset(final.BLCA.matrix,select = final.BLCA.final_info$person)
sum(colnames(final.BLCA.matrix) == final.BLCA.final_info$person)

cat.final_info <- rbind(final.BLCA.final_info,KIRC.final_info,KIRP.final_info,PRAD.final_info)
cat.matrix <- cbind(final.BLCA.matrix,KIRC.matrix,KIRP.matrix,PRAD.matrix)
cat.matrix_M <- log2(cat.matrix / (1 - cat.matrix))
sum(is.na(cat.matrix))

##start to test other GEO datasets
GSE52955_matrix <- fread("03_study_GEO/GSE52955_urinary_cancers/GSE52955_beta_matrix.txt",header = T,sep = "\t")
setkey(GSE52955_matrix,ID_REF)
GSE52955_matrix <- GSE52955_matrix[selected_probes]
GSE52955_matrix$ID_REF <- NULL
GSE52955_matrix_M <- log2(GSE52955_matrix / (1 - GSE52955_matrix))
GSE52955_info <- fread("GSE52955_urinary_cancers/GSE52955_metadata.csv",sep = ",",header = T)
GSE52955_info$final.group <- ifelse(GSE52955_info$group == "Prostate Tumor","PRAD.tumor","others")
sum(colnames(GSE52955_matrix) == GSE52955_info$ID)
df <- as.data.table(t(as.matrix(cat.matrix_M)))
df$class <- factor(cat.final_info$type,levels = c("PRAD.tumor","others"))
colnames(df) <- c(selected_probes,"class")
df.train <- df
table(df.train$class)
df.validate <- as.data.table(t(as.matrix(GSE52955_matrix_M)))
df.validate$class <- factor(GSE52955_info$final.group,levels = c("PRAD.tumor","others"))
colnames(df.validate) <- c(selected_probes,"class")
fit.logit <- glm(as.factor(class)~., data=df.train, family=binomial(),maxit = 25)
summary(fit.logit)
logit.fit.reduced <- step(fit.logit)
summary(logit.fit.reduced)
prob <- predict(logit.fit.reduced, df.validate, type="response")
logit.pred <- factor(prob > .5, levels=c(FALSE, TRUE), labels=c("PRAD.tumor","others"))
logit.perf <- table(df.validate$class, logit.pred, dnn=c("Actual", "Predicted"))
logit.perf
roc_result <- roc(prob,factor(df.validate$class,levels = c("PRAD.tumor","others"),labels = c(0,1)))
plot(roc_result,col="black")
auc(roc_result) #1
```
